# Germ cell quantification in human fetal and prepubertal testis tissues: a comparison of current methodologies

**DOI:** 10.1530/RAF-24-0116

**Published:** 2025-02-07

**Authors:** Emma Kearney, David Greenald, Gabriele Matilionyte, Sheila Lane, Melissa D Tharmalingam, Jill Davies, Jan-Bernd Stukenborg, Grace Forsyth, Rod T Mitchell

**Affiliations:** ^1^ Centre for Reproductive Health, Institute of Regeneration and Repair, University of Edinburgh, Edinburgh, United Kingdom; ^2^ Department of Paediatric Oncology and Haematology, Children’s Hospital Oxford, Oxford University Hospitals NHS Foundation Trust, Oxford, United Kingdom; ^3^ KK Women’s and Children’s Hospital, Singapore; ^4^ Oxford Cell and Tissue Biobank, Children’s Hospital Oxford, Oxford University Hospitals NHS Foundation Trust, Oxford, United Kingdom; ^5^ NORDFERTIL Research Lab Stockholm, Childhood Cancer Research Unit, Department of Women’s and Children’s Health, Karolinska Institutet and Karolinska University Hospital, Solna, Sweden; ^6^ NORDFERTIL Research Lab Uppsala, Department of Organismal Biology, Uppsala University, Uppsala, Sweden; ^7^ Royal Hospital for Children and Young People, Edinburgh, United Kingdom

**Keywords:** human, testis, cisplatin, carboplatin, prepubertal, fetal, fertility, germ cells, gonocytes, spermatogonia, cell quantification

## Abstract

**Abstract:**

Methods to quantify germ cell number in human immature testicular tissues are essential to evaluate the impact of chemotherapy exposures and to optimise cryopreservation protocols used in fertility preservation for prepubertal boys. Established quantification methods rely on the presence of round tubules within the tissue. However, round tubular cross sections are limited in human prepubertal testicular tissues, especially when using *in vitro* culture. We aimed to assess whether an alternative method of germ cell quantification would provide similar results to recently established methods, without the requirement for round tubules. Human testicular samples included fetal tissue (exposed *in vitro* to cisplatin, carboplatin or control) or prepubertal tissue (fresh, cryopreserved, fresh *in vitro* cultured or cryopreserved *in vitro* cultured). Immunofluorescence assessed AP2γ (gonocytes) and MAGE-A4 ((pre)spermatogonia) expression. Germ cells were quantified by tubular germ cell density (Method 1), which was compared to methods that require round tubules, including spermatogonial number per round tubular cross section (S/T) (Method 2), fertility index (Method 3) and round tubular germ cell density (Method 4). A correlation analysis between methods was performed. Method 1 is strongly and significantly correlated with Method 2 (*r* = 0.838, *P* < 0.0001; *r* = 0.833, *P* < 0.0001), Method 3 (*r* = 0.752, *P* < 0.001; *r* = 0.802, *P* < 0.0001) and Method 4 (*r* = 0.863, *P* < 0.0001; *r* = 0.914, *P* < 0.0001) for fetal and prepubertal tissues, respectively. Given that Method 1 assess tubules irrespective of shape, it may increase the total number of germ cells available for quantification, validating its use for quantification of human testicular tissue samples where the amount of tissue or presence of round tubules is limited.

**Lay summary:**

Chemotherapy can damage cells in the testicles that are required to make sperm, often leading to infertility in males. While options to preserve fertility in adult males are available, there are no established methods for young boys. To investigate how chemotherapy damages these cells and to explore approaches to preserve fertility, we require methods to count the number of cells that can develop into sperm. Existing counting methods involve only counting some of the cells in the tissue, but in young boys, it is often necessary to count all of the cells because the amount of tissue is limited. To overcome this, we counted cells in small pieces of human fetal and prepubertal testicles using an alternative method, which allows all cells to be counted. We found similar results using our method compared to three existing methods, making our method useful for counting cells in fetal and prepubertal testicle samples.

## Background

Spermatogenesis commences post-puberty, yet the germ cell number and composition within the testis undergoes dynamic changes from prenatal development through to the onset of puberty (Ntemou *et al.* 2019). During the fetal stage, the germ cell populations comprise gonocytes and (pre)spermatogonia, which express AP2γ and MAGE-A4, respectively (Pauls *et al.* 2006, Mitchell *et al.* 2008, Tharmalingam *et al.* 2020). In infancy, the remaining gonocytes undergo differentiation into spermatogonia, and throughout childhood, spermatogonia constitute the predominant germ cell population within the testis (Altman *et al.* 2014, Ntemou *et al.* 2019). Therefore, the survival and functionality of spermatogonia during childhood is essential for future fertility.

Exposure to chemotherapy during childhood can result in partial or complete loss of spermatogonia, with different chemotherapy drugs associated with a higher risk of testicular damage (Lopes *et al.* 2021). Notably, alkylating agents have been described amongst the most gonadotoxic compounds (Green *et al.* 2014, Poganitsch-Korhonen *et al.* 2017, Stukenborg *et al.* 2018, Tharmalingam *et al.* 2020). Furthermore, due to the frequent administration of chemotherapeutic drugs in combination, the relative gonadotoxicity of individual agents is challenging to define (Allen *et al.* 2018, Smart *et al.* 2018). Therefore, accurate germ cell quantification methods are crucial for studying how exposure to chemotherapy agents used in paediatric oncology can impair male fertility.

Currently, fertility preservation for prepubertal males is focused on cryopreservation of testicular tissue before treatment and the subsequent transplantation of tissue or cells or *in vitro* maturation of germ cells (Picton *et al.* 2015, Wyns *et al.* 2021, Duffin *et al.* 2024). However, successful restoration of fertility using cryopreserved testicular tissue has not yet been achieved in humans (Allen *et al.* 2018). As cryopreservation is an experimental approach, with no standardised protocol, knowledge of germ cell number is essential to assess the effects of tissue freezing and thawing on germ cell survival (Goossens *et al.* 2020, Duffin *et al.* 2024). This will enable researchers to adapt freezing protocols to ensure tissue viability is maintained. In addition, knowledge of germ cell number in stored tissue samples could hold potential to personalise future fertility restoration approaches (Funke *et al.* 2021, Lahtinen *et al.* 2024).

The methods to quantify germ cell number in testicular tissue samples vary between studies (Poganitsch-Korhonen *et al.* 2017, Stukenborg *et al.* 2018, Portela *et al.* 2019, Kurek *et al.* 2021, Medrano *et al.* 2021, Benninghoven-Frey *et al.* 2022, Feraille *et al.* 2023, Matilionyte *et al.* 2023, Lahtinen *et al.* 2024). Recent methods aimed at standardising germ cell quantification rely on the presence of a sufficient number of round tubules (Steinberger & Tjioe 1968, Funke *et al.* 2021). However, human fetal and prepubertal testis tissues often have few round tubules, leading to exclusion of samples and potentially reducing the statistical power of the experiment. This limitation is also relevant to experimental procedures, such as *in vitro* culture conditions, where small fragments of tissue are used and round tubules are rare (Jørgensen *et al.* 2014, Portela *et al.* 2019, Aden *et al.* 2023). Improving germ cell quantification methods to accommodate samples with fewer round tubules would enhance the inclusivity of testicular tissue samples for analysis, which is important given the limited availability of human samples for experimental use.

In the present study, we utilised human fetal testicular tissue, a surrogate model for the prepubertal testis (Matilionyte *et al.* 2023), which had previously been exposed to chemotherapy drugs. In addition, fresh and cryopreserved prepubertal testicular tissue, with or without *in vitro* culture, was analysed. The aim of this study was to compare a germ cell quantification method that does not require the presence of round tubules with three currently utilised quantification methods that rely on the presence of a sufficient number of round tubules.

## Materials and methods

### Study approval

Ethical approval for the use of human fetal testicular tissue for research was obtained from the South East Scotland Research Ethics Committee (REC Ref 13/SS0145). Written informed consent to donate tissue for research was obtained from pregnant women. The collection and use of human prepubertal testicular tissue were ethically approved by the South East Scotland Research Ethics Committee (13/SS0145) and the Oxford University Hospitals NHS Foundation Trust (2016/0140). Written informed consent was obtained from patients and/or their parents/guardians, as appropriate.

### Tissue collection

#### Human fetal testicular tissue

Human testes were obtained from fetuses following medical or surgical termination of pregnancy during the second trimester (14–22 gestational weeks; total *n* = 6). Terminations due to known fetal abnormalities were excluded. The gestational age of the fetuses was determined initially through ultrasound and then by direct measurement of foot length. PCR for the male-specific sex-determining region Y (SRY) gene was performed to confirm the sex of the fetus. Samples were placed in PBS and transported to Edinburgh research facilities.

#### Human prepubertal testicular tissue

Testicular tissue was obtained from prepubertal patients (total *n* = 6; aged 1–12 years) undergoing biopsy for fertility preservation, who had received a cancer diagnosis but not yet undergone chemotherapy treatment. From the size of the biopsies obtained, around 10–20% of the sample was used for research purposes. Samples were placed in Nutristem® hSEC XF Medium (Biological Industries, Israel) supplemented with 1% penicillin/streptomycin (Sigma-Aldrich, USA) and transported from the operating theatre on ice to Edinburgh research facilities and subsequently processed within 24 h.

### Tissue preparation and processing

**Figure 1:**
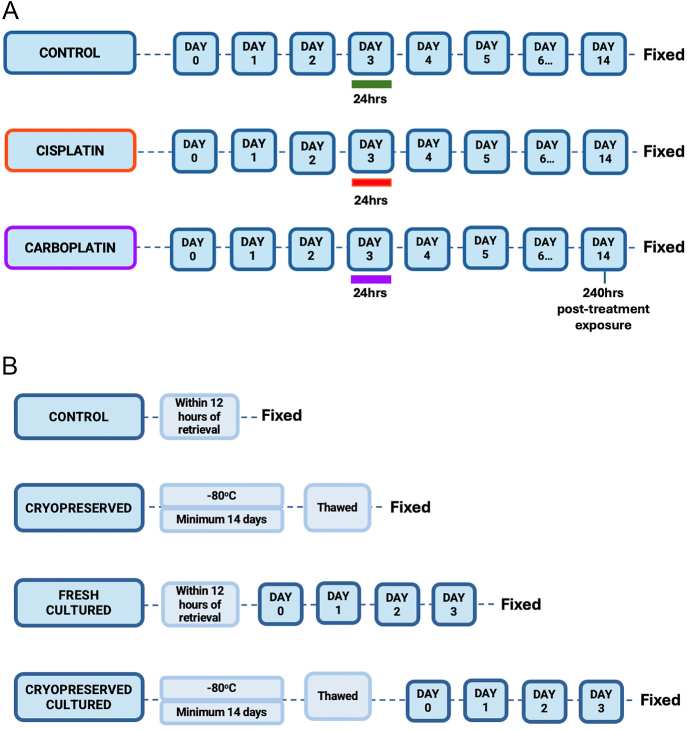
Schematic representation of tissue culture experiments for fetal (A) and prepubertal (B) tissues. Fetal testicular tissue (A) was exposed to medium containing ddH20 (green line), cisplatin (red line) or carboplatin (purple line) for 24 h and analysed at 240 h post-treatment exposure. Prepubertal testicular tissue (B) was either fresh or cryopreserved for a minimum of 14 days, with or without *in vitro* culture.

Methods to quantify germ cells were compared in several experimental conditions using either human fetal or prepubertal tissues. The experimental groups are summarised in Fig. 1.

#### Human fetal testicular tissue

For the first experiment, human fetal tissue was cut (∼1 mm^3^) and cultured in hanging drops consisting of 30 μL droplets of culture media at 37 °C in 5% CO_2_ (Jørgensen *et al.* 2014). These cultures were performed for a previously published study, in which Method 1 was used to quantify germ cells (Tharmalingam *et al.* 2020). The culture medium consisted of alpha MEM (Lonza, Switzerland), 1% penicillin/streptomycin, 1% non-essential amino acids, 2 mM l-glutamine, 2 mM sodium pyruvate and 11% insulin–transferrin–selenium and was supplemented with 10% fetal bovine serum (FBS; all Sigma-Aldrich, USA). The fetal tissue pieces were cultured for 24–72 h before being exposed to a medium consisting of a vehicle control (ddH_2_0), cisplatin (0.5 μg/mL; Sigma-Aldrich, USA) or carboplatin (5 μg/mL; Sigma-Aldrich, USA) on day 3 for 24 h. The tissue pieces were then cultured in drug-free medium and analysed at 240 h post-chemotherapy exposure (14-day culture). At the end of the experiment, tissue sections were fixed in Bouin’s fluid (Clin-Tech) for 1 h, transferred to 70% ethanol and embedded in paraffin blocks. Sections were cut into 5-μm sections and used for immunofluorescence. For each experimental group, two sections approximately a third from the beginning and end of the tissue block, from two replicate tissue pieces were analysed.

#### Human prepubertal testicular tissue

##### Control (fresh)

For each patient, freshly isolated fragments of testicular tissue were retained to make up the control experimental group. These tissue pieces were fixed in Bouin’s fluid (Clin-Tech) for 1 h, transferred to 70% ethanol and embedded in paraffin blocks.

##### Cryopreserved

To prepare the cryopreserved group, small fragments (∼1 mm^3^) of testicular tissue from each patient were cryopreserved. Fragments were placed in 2 mL cryovials (Corning, USA) with 1 mL cryoprotectant media consisting of DMEM (Life Technologies, USA) supplemented with 10% DMSO (1.28 M; Sigma-Aldrich) and 15% FBS (GIBCO) at 4 °C. Cryovials were transferred into a pre-cooled Mr Frosty container (Thermo Fisher Scientific, USA) and cooled overnight (approximately −1 °C/min) in a −80 °C ULT freezer. After being held at −80 °C, cryovials were placed into liquid nitrogen for a minimum of two weeks. To thaw tissue, cryovials were placed into a 37 °C water bath until thawed and subsequently washed in PBS three times for 5 min each. Then, the tissue samples were fixed in Bouin’s fluid (Clin-Tech) for 1 h, transferred to 70% ethanol and embedded in paraffin blocks.

##### Fresh cultured

For the *in vitro* fresh cultured group, a hanging drop culture system was used, as described above for fetal testicular tissue. Fragments of fresh tissue were cultured for 96 h in 40 μL droplets of culture media containing alpha MEM without l-glutamine (Thermo Fisher) and 10% knockout serum replacement (GIBCO, USA). Dishes were incubated at 37 °C and 5% CO_2_ for 96 h, with media replaced every 48 h. At the end of 96 h, fragments were directly fixed in Bouin’s fluid (Clin-Tech) for 1 h, transferred to 70% ethanol and embedded in paraffin blocks.

##### Cryopreserved cultured

For the *in vitro* cultured group, using cryopreserved tissue, frozen–thawed tissue was placed into hanging drop culture as described for fresh cultured tissue, followed by fixation and paraffin embedding as described above.

For the four experimental conditions, paraffin blocks were cut into 5-μm sections and placed on slides. For each experimental group, two sections approximately a third from the beginning and end of the tissue block, from two replicate tissue pieces were analysed.

### Immunofluorescence

#### Human fetal testicular tissue

Immunofluorescence for AP2γ (gonocytes) and MAGE-A4 ((pre)spermatogonia) was previously performed as described by Tharmalingam *et al.* (2020). Details of antibodies and dilutions are provided in Table 1. In short, tissue sections were dewaxed by incubating in xylene, passed through decreasing alcohol series (100–70%) for the rehydration process and then washed with distilled water. Heat-induced antigen retrieval was achieved by pressure cooking in citrate buffer (0.01 M). Slides were then incubated in 3% hydrogen peroxide in methanol to prevent endogenous peroxide activity and non-specific sites were blocked with blocking agent consisting of 20% normal goat serum in Tris-buffered saline (TBS) with 5 g BSA. Sections were incubated overnight at 4 °C with primary antibody diluted in blocking solution. Tissue sections were then washed in TBS and incubated for 30 min with the appropriate peroxidase-conjugated secondary antibody to detect the primary antibody. A signal amplification kit (Opal™, Akoya Biosciences, USA) was used for visualisation at 1:100 for 10 min. If a subsequent primary antibody was added, a further antigen retrieval step was carried out and detection steps were repeated using a different fluorophore. Cell nuclei were counterstained using Hoechst (Thermo Fisher Scientific) at 1:2000 dilution in TBS. Slides were mounted with Permafluor (Lab Vision™, Thermo Scientific, USA). Tiled images of tissue sections were acquired using an LSM 780 (Carl Zeiss, Germany) confocal microscope at 20× magnification.

**Table 1:** Summary of the immunofluorescence protocol for fetal and prepubertal tissues.

Antibody	Cell type detection	Dilution	Cat no.	Supplier
Primary				
MAGE-A4	(Pre)spermatogonia (fetal); spermatogonia (PPB)	1:200	N/A	Gifted from Giulio Spagnoli
AP2γ	Gonocytes (fetal)	1:200	ssc-12762	Santa Cruz Biotechnology
Secondary				
Goat anti-mouse peroxidase	N/A	1:200	P0447	Dako

#### Human prepubertal testicular tissue

For human prepubertal testicular tissue, immunofluorescence for MAGE-A4 was performed as described above. Details of antibodies and dilutions are provided in Table 1.

### Cell quantification

Germ cells were identified based on positive expression of MAGE-A4 and/or AP2γ and quantified using Qupath v0.4.3 (https://github.com/qupath/qupath/releases/tag/v0.4.3; Bankhead *et al.* 2017). Image analysis was conducted with the assessor blinded to experimental groups. The testicular region of interest and individual tubules were manually outlined using Hoechst staining as a guide and the ‘*brush*’ tool, allowing for the determination of total number of tubules and the calculation of the tubular area (mm^2^). The identification of round tubules was performed by measuring the longest and shortest diameters of each tubule using the ‘*grid*’ tool, with a ratio of <1.5 defining round tubules. Positively stained germ cells were manually counted within the annotated regions using the ‘*point*’ tool, clicking on each positively stained cell. Gonocytes were identified by their nuclear staining for AP2γ, while (pre)spermatogonia were identified by their cytoplasmic staining for MAGE-A4. Individual tubules at the periphery of tissue sections were excluded if their basement membrane was incomplete, ensuring that whole intact tubules were included in the analysis.

All data, including the number of tubules (round and total) and tubular area (round and total), were extracted from QuPath using the data output function ‘*show detection measurements*’. For each section from all samples (fetus/patient), the following four formulas were used for germ cell quantification:

Method 1:

$$\text{Tubular}\;\text{germ}\;\text{cell}\;\text{density}=\frac{\;\text{the}\;\text{number}\;\text{of}\;\text{spermatogonia}}{\text{total}\;\text{tubular}\;\text{area}\;\left({\text{mm}}^{2}\right)}$$

Method 2:

$$\text{Spermatogonial}\;\text{number}\;\text{per}\;\text{round}\;\text{tubular}\;\text{cross}\;\text{section}\;\left(S/T\right)=\frac{\text{the}\;\text{number}\;\text{of}\;\text{spermatogonia}\;\text{in}\;\text{round}\;\text{tubules}}{\text{the}\;\text{number}\;\text{of}\;\text{round}\;\text{tubules}}$$

Method 3:

$$\text{Fertility}\;\text{index}\;\left(\text{FI}\right)=\frac{\text{the}\;\text{number}\;\text{of}\;\text{round}\;\text{tubules}\;\text{with}\;\text{spermatogonia}}{\text{the}\;\text{number}\;\text{of}\;\text{round}\;\text{tubules}}\times 100$$

Method 4:

$$\text{Round}\;\text{tubular}\;\text{germ}\;\text{cell}\;\text{density}=\frac{\;\text{the}\;\text{number}\;\text{of}\;\text{spermatogonia}\;\text{in}\;\text{round}\;\text{tubules}}{\text{round}\;\;\text{tubular}\;\text{area}\;\left({\text{mm}}^{2}\right)}$$

### Statistical analysis

All data were analysed using GraphPad Prism version 10 (GraphPad Software, USA; https://www.graphpad.com/updates/prism-1000-release-notes). Comparisons between the experimental conditions were examined for statistical significance using a two-way analysis of variance (ANOVA), followed by Tukey’s post hoc test for normally distributed data. For data where normality was not shown, the Kruskal–Wallis non-parametric test, followed by Dunn’s multiple comparisons test was used. All data are presented as mean ± standard deviation (SD). To evaluate the correlation between Method 1 and the other three quantification methods, Pearson’s correlation coefficient analysis was used. For all tests performed, statistical significance was considered where *P* < 0.05.

## Results

### Determining the effects of cisplatin and carboplatin on germ cell number in human fetal testicular tissue using four different quantification methods

**Figure 2:**
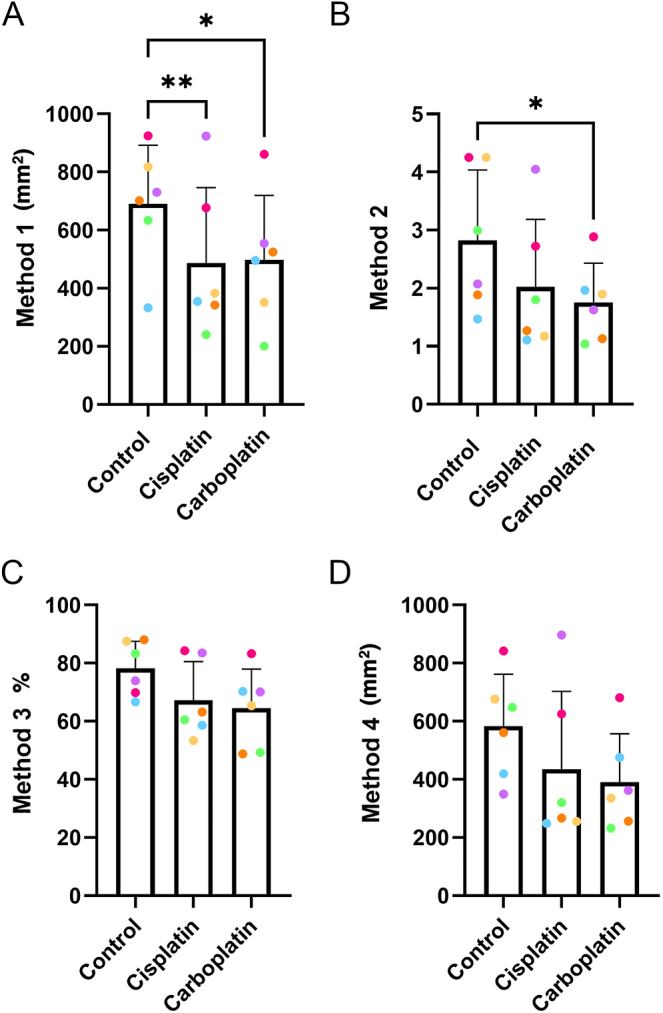
Different methods for quantifying the effects of exposure to cisplatin or carboplatin on germ cell number in human fetal testicular tissue. Germ cells were quantified by Methods 1 (A), 2 (B), 3 (C) and 4 (D), 240 h post-exposure to control, cisplatin or carboplatin. Data analysed using two-way ANOVA (A, B, C) or Kruskal–Wallis test (D) (*P < 0.05, ** *P* < 0.01) and presented as mean ± SD. Each set of data points presented in the same colour represents the mean value for all fragments obtained from an individual fetus (*n* = 6).

To compare the different quantification methods in human fetal testis, we used tissues exposed to vehicle or platinum-based chemotherapy agents. Immunofluorescence staining was performed for MAGE-A4 ((pre)-spermatogonia) and AP2γ (gonocytes) and cells were quantified. At 240 h post-treatment exposure, there was a significant reduction in germ cell number in both cisplatin- (486.7 ± 259.8 vs 690.2 ± 201.9 germ cells per mm^2^, *P* = 0.0079) and carboplatin-exposed tissue (497.8 ± 221.8 vs 690.2 ± 201.9, *P* = 0.0150), compared with control, when quantification was carried out using Method 1 (Fig. 2A). Quantification of germ cells using Method 2 also revealed a significant reduction in the carboplatin-exposed tissue compared to the control-exposed testicular tissue (1.76 ± 0.67 vs 2.82 ± 1.22 germ cells per mm^2^, *P* = 0.02; Fig. 2B). However, with Method 2, a non-significant reduction in germ cell number in cisplatin-exposed tissue was observed when compared to control (2.02 ± 1.16 vs 2.82 ± 1.22 germ cells per mm^2^, *P* = 0.0978; Fig. 2B). Furthermore, when tissue sections were quantified using Methods 3 and 4, respectively, a similar trend toward a reduction in germ cells was observed, but this did not reach statistical significance for samples exposed to cisplatin (67.28 ± 13.29 vs 78.23 ± 9.29 germ cells per mm^2^, *P* = 0.3101; Fig. 2C) (435.7 ± 267.7 vs 582.7 ± 179.8 germ cells per mm^2^, *P* = 0.3505; Fig. 2D) or carboplatin (64.54 ± 13.40 vs 78.23 ± 9.287 germ cells per mm^2^, *P* = 0.1344; Fig. 2C) (390.4 ± 166.7 vs 582.7 ± 179.8 germ cells per mm^2^, *P* = 0.390; Fig. 2D), when compared with control.

### Correlation between methods to quantify the effects of cisplatin and carboplatin on germ cell number in fetal testicular tissue

**Figure 3:**
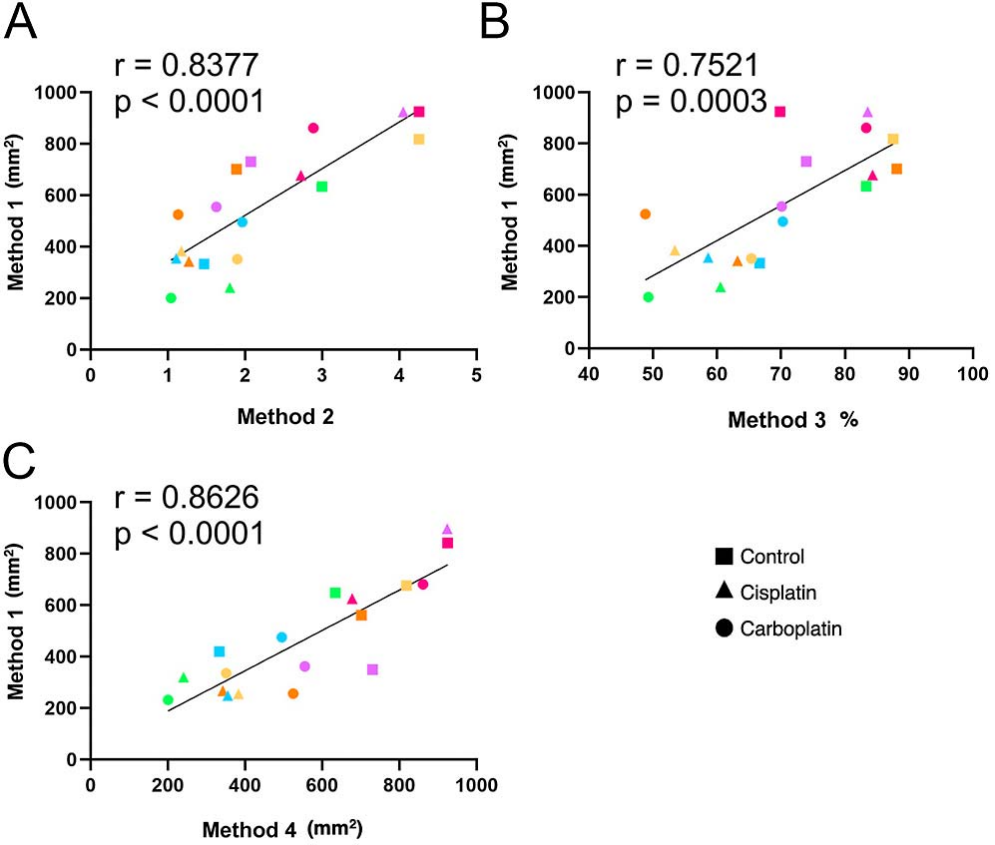
Correlation analysis between Method 1 and Methods 2 (A), 3 (B) and 4 (C) in vehicle control, cisplatin and carboplatin treatment groups. Each set of data points presented in the same colour represents the mean value for all fragments obtained from an individual fetus (*n* = 6), while the data point shape signifies different treatment groups. The solid line represents the best fit for the correlation between the variables being compared. Pearson’s correlation coefficient, *r*.

**Figure 4:**
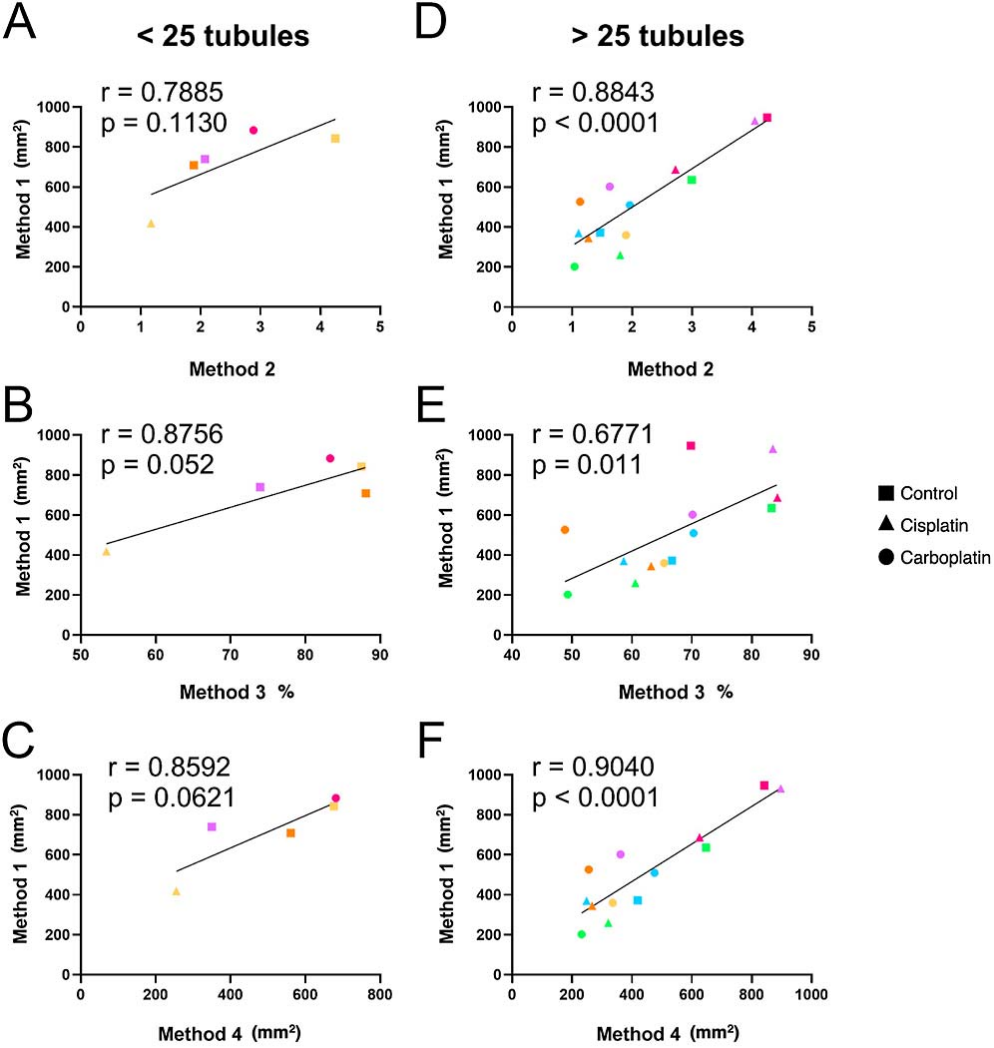
Correlation analysis between Method 1 and Methods 2 (A, D), 3 (B, E) and 4 (C, F) in vehicle control, cisplatin and carboplatin treatment groups. Samples that had <25 (A–C) and >25 (DF) round tubular cross sections were analysed separately. Data points presented in the same colour represent the mean value for all fragments obtained from an individual fetus (*n* = 6), while the data point shape represents different treatment groups. The solid line represents the best fit for the correlation between the variables being compared. Pearson’s correlation coefficient, *r*.

Given that a similar trend toward a reduction in germ cells was observed in cisplatin- and carboplatin-exposed testicular tissue across the four quantification methods, Pearson’s correlation analysis was performed. Method 1 was compared with Methods 2, 3 and 4, to assess the strength of the relationship between methods. A significant and strong correlation was observed when comparing Method 1 with Method 2 (*r* = 0.8377, *P* < 0.0001; Fig. 3A), 3 (*r* = 0.7521, *P* = 0.0003; Fig. 3B) and 4 (*r* = 0.8626, *P* < 0.0001; Fig. 3C), for vehicle control, cisplatin- and carboplatin-treated groups.

Since the tissue composition and spatial location of germ cells are highly variable in the fetal and prepubertal testis, samples which had <25 and ≥25 round tubules were analysed separately to establish if correlations between methods are maintained when smaller sections of testicular tissue are examined. In samples which had <25 round tubules, a strong but non-significant correlation was observed when Method 1 was compared with Method 2 (*r* = 0.7885, *P* = 0.1130; Fig. 4A), Method 3 (*r* = 0.8756, *P* = 0.052; Fig. 4B) and Method 4 (*r* = 0.8592, *P* = 0.0621; Fig. 4C), for vehicle control, cisplatin- and carboplatin-treated groups. However, in samples which had ≥25 round tubules, Pearson’s correlation revealed a strong and significant correlation when Method 1 was compared with Method 2 (*r* = 0.8843, *P* < 0.0001; Fig. 4D), Method 3 (*r* = 0.6771, *P* = 0.011; Fig. 4E) and Method 4 (*r* = 0.9040, *P* < 0.0001; Fig. 4F).

### Determining the effects of cryopreservation on germ cell number in human prepubertal testicular tissue using four different quantification methods

**Figure 5:**
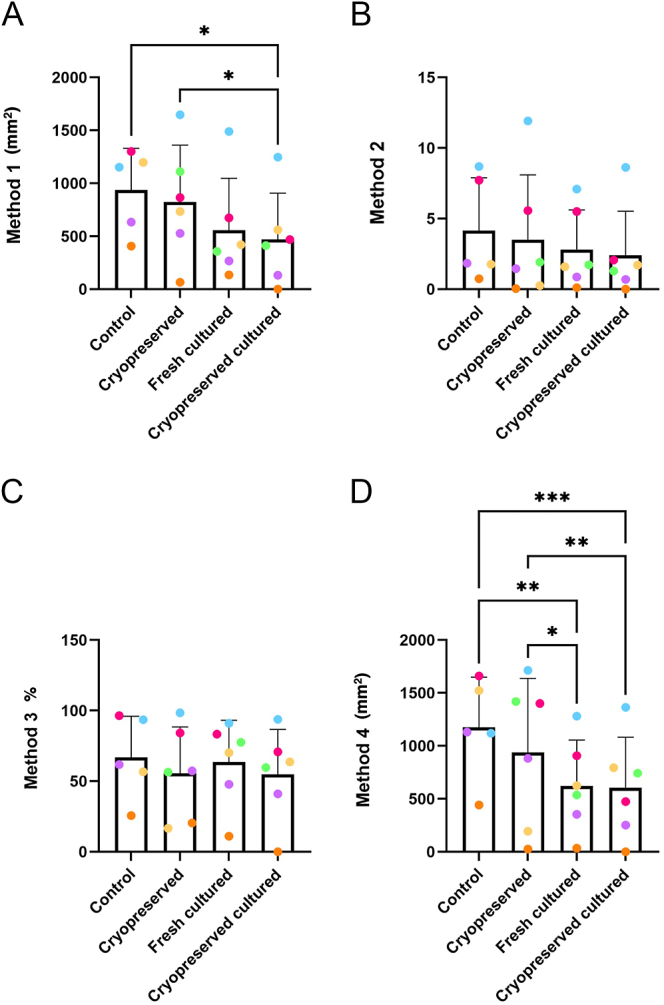
Different methods for quantifying the effects of cryopreservation on germ cell number in human prepubertal testicular tissue. Experimental groups included control, cryopreserved, fresh cultured or cryopreserved cultured. Germ cells were quantified by Methods 1 (A), 2 (B), 3 (C) and 4 (D). Data analysed using two-way ANOVA (A, C, D) or Kruskal–Wallis test (B) (*P < 0.05, ** *P* < 0.01, *** *P* < 0.001) and presented as mean ± SD. Each set of data points presented in the same colour represents the mean value for all fragments obtained from an individual patient (*n* = 5–6).

Quantification methods revealed similar trends and close correlations for assessing germ cell number in human fetal tissue exposed to platinum-based chemotherapeutics. Therefore, we evaluated how the various quantification methods compare to detect changes in germ cell number, using human testicular tissue from prepubertal patients. Samples were subjected to cryopreservation and/or different culture conditions. Immunofluorescence staining for MAGE-A4 was performed to identify spermatogonia. Quantification using Method 1 detected a significant difference in germ cell number between cryopreserved tissue before and after culture (825.3 ± 535.3 vs 470.4 ± 436.3 germ cells per mm^2^, *P* = 0.0376) (Fig. 5A). When quantification was performed using Method 2 or Method 3, no statistical differences were observed between any of the experimental conditions (Fig. 5B and C). However, when samples were quantified using Method 4, significant differences were detected when control tissue was compared with fresh cultured tissue (1,175 ± 473.7 vs 623.2 ± 433.3 germ cells per mm^2^, *P* = 0.0012) or cryopreserved cultured tissue (1,175 ± 473.7 vs 604.5 ± 477.3 germ cells per mm^2^, *P* = 0.0002) and when cryopreserved tissue was compared with fresh cultured tissue (939.4 ± 697.8 vs 623.2 ± 433.3 germ cells per mm^2^, *P* = 0.0174) or cryopreserved cultured tissue (939.4 ± 697.8 vs 604.5 ± 477.3 germ cells per mm^2^, *P* = 0.0032) (Fig. 5D).

### Correlation between methods to quantify the effects of cryopreservation and/or *in vitro* culture on germ cell number in prepubertal testicular tissue

**Figure 6:**
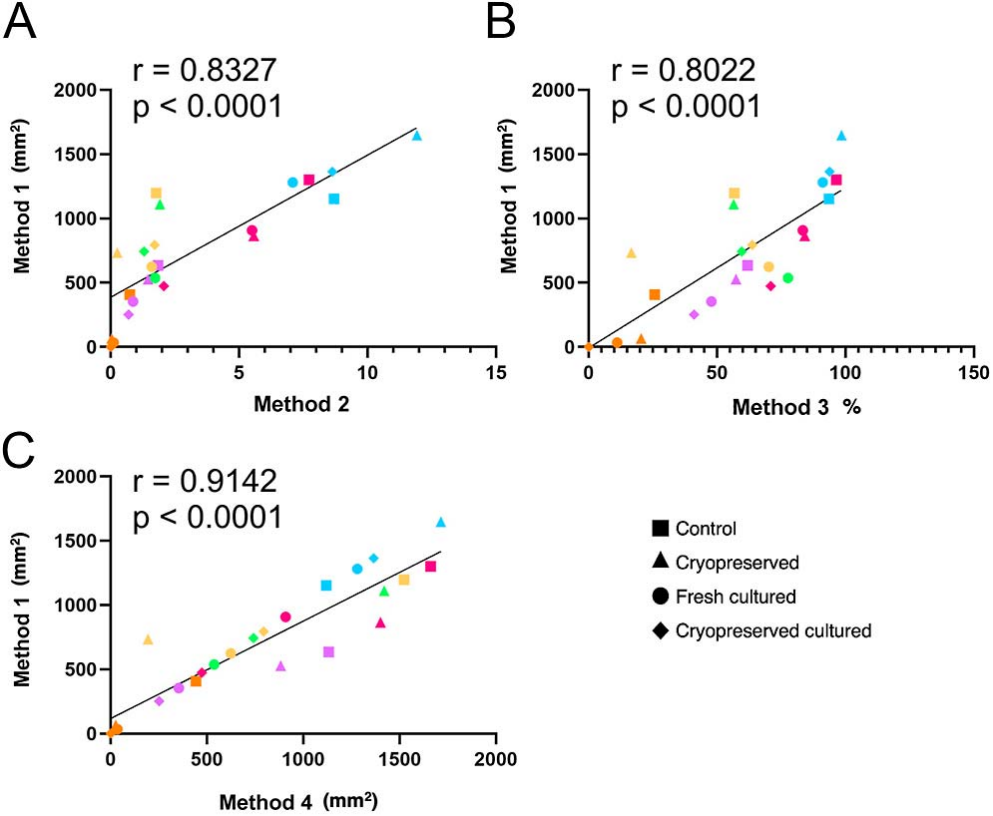
Correlation analysis between Method 1 and Methods 2 (A), 3 (B) and 4 (C) in control, cryopreserved, fresh cultured and cryopreserved cultured tissue. Each set of data points presented in the same colour represent the mean value for all fragments obtained from an individual patient (*n* = 5–6), while the data point shape signifies different culture conditions. The solid line represents the best fit for the correlation between the variables being compared. Pearson’s correlation coefficient, *r*.

Similar trends in the data were observed across the four quantification methods when determining the effects of cryopreservation and culture on germ cell number. Therefore, Pearson’s correlation analysis was performed to further evaluate the strength of the relationship between the various quantification methods. Pearson’s correlation analysis indicated that Method 1 was strongly and significantly correlated with Method 2 (*r* = 0.8327, *P* < 0.0001; Fig. 6A), Method 3 (*r* = 0.8022, *P* < 0.0001; Fig. 6B) and Method 4 (*r* = 0.9142, *P* < 0.0001; Fig. 6C), for all the experimental groups.

**Figure 7:**
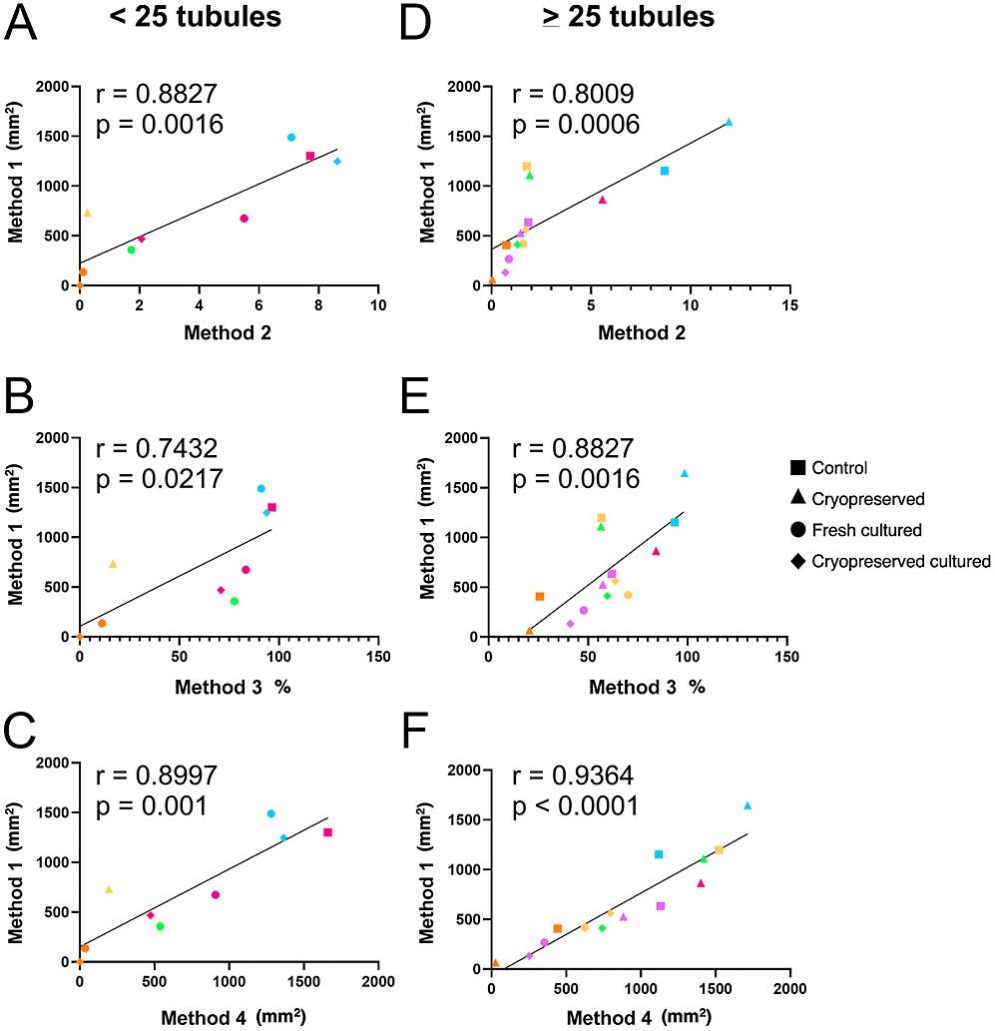
Correlation analysis between Method 1 and Methods 2 (A, D), 3, (B, E) and 4 (C, F) in control, cryopreserved, fresh cultured and cryopreserved cultured groups. Samples that had <25 (A–C) and >25 (D–F) round tubular cross sections were analysed separately. Data points presented in the same colour represent the mean value for all fragments obtained from an individual patient (*n* = 5–6), while the data point shape signifies different experimental groups. The solid line represents the best fit for the correlation between the variables being compared. Pearson’s correlation coefficient, *r*.

To assess how the quantification methods compare when only a small part of prepubertal testicular tissue is examined, samples were grouped and analysed together depending on whether they had <25 or ≥25 round tubules. In samples with <25 round tubules, Pearson’s correlation analysis indicated that Method 1 was significantly and strongly correlated with Method 2 (*r* = 0.8827, *P* = 0.0016; Fig. 7A), Method 3 (*r* = 0.7432, *P* = 0.0217; Fig. 7B) and Method 4 (*r* = 0.8997, *P* < 0.001; Fig. 7C). Likewise, in samples with ≥25 round tubules, Method 1 was both significantly and strongly correlated with Method 2 (*r* = 0.8009, *P* = 0.0006; Fig. 7D), Method 3 (*r* = 0.8827, *P* = 0.0016; Fig. 7E) and Method 4 (*r* = 0.9364, *P* ≤ 0.0001; Fig. 7F).

## Discussion

Methods of germ cell quantification, which provide an accurate and reproducible estimation of spermatogonial numbers, are required for investigating the effects of various factors, including drug exposure and cryopreservation methods, on the prepubertal testis. In this study, we utilised human fetal testicular tissue previously exposed to chemotherapy agents, and fresh or cryopreserved prepubertal testicular tissue, with or without *in vitro* culture, and assessed the germ cell number. We aimed to determine whether an alternative quantification method, which does not rely on the presence of round tubules, can provide results comparable to other germ cell quantification methods, which require the presence of round tubules (Poganitsch-Korhonen *et al.* 2017, Stukenborg *et al.* 2018, Kurek *et al.* 2021, Medrano *et al.* 2021, Benninghoven-Frey *et al.* 2022, Feraille *et al.* 2023).

Results from this study demonstrate that quantification using Method 1, which includes all seminiferous tubules in the analysis, is strongly and significantly correlated with quantification methods, which exclusively assess round tubules. This positive correlation validates Method 1 as a suitable alternative approach for germ cell quantification, maintaining consistency with other approaches. Moreover, as Method 1 has potential to include more tubules and germ cells in the analysis, it may be more sensitive to detecting biological variability within testicular tissue samples when the amount of material available is limited or round tubules are rare. This is particularly relevant for studies involving human fetal and prepubertal testis tissue, especially following *in vitro* culture.

The most widely reported germ cell quantification methods for prepubertal testicular tissue include S/T (Method 2) and FI (Method 3) (Cortes 1990, Poganitsch-Korhonen *et al.* 2017, Stukenborg *et al.* 2018, Benninghoven-Frey *et al.* 2022, Feraille *et al.* 2023, Lahtinen *et al.* 2024). In this study, unlike Method 1, Methods 2 and 3 did not detect statistically significant differences between treatment groups, with the exception of carboplatin- vs control-exposed tissue quantified with Method 2. The lack of significant differences between experimental groups with Methods 2 and 3 may arise from reduced statistical power attributable to the analysis of fewer tubules and germ cells compared to Method 1. The observed reduction in germ cell number in response to cisplatin exposure is consistent with preclinical and clinical evidence of cisplatin-induced testicular damage reported in the literature (Romerius *et al.* 2011, Chow *et al.* 2016, Smart *et al.* 2018, Yaman & Topcu-Tarladacalisir 2018, Allen *et al.* 2020, Tharmalingam *et al.* 2020).

Quantification methods, which focus on round tubules, are reliant on sectioning the tissue perpendicular to the longitudinal axis of the tubule. This orientation ensures that the researcher is observing a cross section of the tubule, providing an aspect of consistency in the analysis of germ cells within the tubules. However, the orientation of the tubules in human prepubertal tissues often results in relatively few round tubules per tissue cross section. Experimental procedures, such as organotypic culture systems used in this study, involve small volumes of tissue (∼1 mm^3^). Consequently, the number of round tubules is restricted by the small size of tissue samples. Moreover, the longitudinal growth of seminiferous tubules during post-natal life in primates can lead to spatial variation in germ cell density within the tubules in prepubertal testicular samples (Albert *et al.* 2010, Funke *et al.* 2021). Therefore, caution is required when extrapolating findings from a limited number of round tubules. To mitigate this potential bias, where feasible, increasing the number of testicular samples will enhance the likelihood of obtaining sufficient round tubules to improve the representativeness of the analysis.

To ensure the validity of results, it is recommended to analyse a minimum of 25 round tubular cross sections per sample (Steinberger & Tjioe 1968). This number has been demonstrated to be sufficient to represent an entire adult testicular biopsy (Steinberger & Tjioe 1968). In their study of six healthy testicular biopsies, the total number of round seminiferous tubules ranged from 56 to >100 per biopsy. However, the prepubertal and fetal testicular tissues often contain few round tubules. Within our study, 28% of fetal and 39% of prepubertal samples did not contain as many as 25 round tubules. Following the convention of analysing a minimum of 25 tubular cross sections would necessitate excluding these samples from the analysis, unless additional sections at least 10–15 µm apart are available. This highlights a limitation in applying quantification Methods 2–4 to fetal and prepubertal testicular tissue, where sample size and tissue availability is already constrained (Portela *et al.* 2019, Matilionyte *et al.* 2022).

Despite this, strong correlations between Method 1 and the other three quantification methods were found in samples with less than 25 tubules, although this did not reach significance for fetal testicular tissues. While quantification Methods 2–4 may provide some insights even when the threshold of 25 tubules cannot be met, the adequacy of analysing less than 25 tubules for an accurate comparison of germ cell number remains uncertain. Given that these findings are based on a limited number of samples, further research is needed to determine a reliable threshold for the number of round tubules required to represent an entire human fetal and prepubertal testicular biopsy. Similarly, while Method 1 has demonstrated good reliability with a minimum total tubular area of 0.015 and 0.003 mm^2^ for fetal and prepubertal tissue, respectively, the lower threshold for tubular area has yet to be determined.

When evaluating germ cell number, it is important to consider the relevance of the quantification method to the research question, particularly in studies focused on future fertility potential. Germ cell quantification Methods 1 and 4, which normalise germ cell counts relative to the tubule area, are advantageous for samples where tubules vary significantly in size. By accounting for instances where a small tubule may contain as many germ cells as a larger tubule, these methods may provide a more precise risk prediction of future male sub/infertility, which is valuable for studies assessing the potential gonadotoxic effects of a variety of treatments and strategies to mitigate such damage.

In contrast to Methods 1, 2 and 4, Method 3, although quick to perform, involves quantification of the percentage of tubules containing germ cells. Despite a strong and significant correlation with Method 1, Method 3 has limited power to detect differences in germ cell number, given variations in germ cell density within each tubule. For instance, a high FI with Method 3 may be associated with a low overall germ cell number if spermatogonia and/or gonocytes are sparsely distributed. While Method 3 is suitable to gain insight into the distribution of germ cells over the testis, irrespective of the total germ cell number, it is crucial that researchers understand the limitations of germ cell quantification methods to ensure accurate interpretation of results. As demonstrated in previous studies, researchers have adopted a combined approach to germ cell quantification, where Method 3 is complemented with Method 2 (Poganitsch-Korhonen *et al.* 2017, Benninghoven-Frey *et al.* 2022, Feraille *et al.* 2023). This approach is necessary to overcome the limitations inherent with relying on Method 3 alone to assess future fertility potential.

Given that testicular tissue for research can be sourced from a diverse range of patients varying in age and reproductive development, the number of germ cells in these samples can differ significantly between patients (Funke *et al.* 2021). A recent study has developed Z-scores as a method of correcting for these variables (Funke *et al.* 2021). Establishing *Z*-scores that can be applied to Method 1 would help control developmental variation by normalising age-dependent differences in germ cell number across tissue samples in studies where tissue is limited or round tubules are rare. Such standardisation would also enable large-scale studies and meta-analyses to be performed. To increase the translational potential of this approach, future research should validate clinically relevant *Z*-score thresholds that correlate with key outcomes, such as the success or failure of fertility preservation strategies or the recovery of spermatogenesis following chemotherapy exposure. By determining the cut-off values, clinicians would be able to improve their counselling of patients, providing better guidance on treatment options and fertility preservation strategies. Moreover, future studies should be aimed at developing automated image analysis techniques to further standardise germ cell quantification. Approaches such as deep learning models (i.e., U-Net) can be trained to perform semantic segmentation to detect/measure tubules and quantify germ cells, thereby reducing human error and increasing the efficacy of germ cell counting.

## Conclusion

This study validates an alternative germ cell quantification method for comparing germ cell number in human fetal and prepubertal testis tissues, demonstrating its applicability across various experimental settings. The strong correlation with other widely used quantification methods highlights the suitability of the method as a standard technique for quantifying germ cells. This method can be employed for assessing germ cell number in human fetal and prepubertal testicular tissue, providing a robust analysis that maximises the inclusion of testicular tissue samples, particularly when the presence of round tubules is limited. This method of germ cell quantification represents a valuable approach for experimental studies aimed at estimating the quality of prepubertal testicular tissue samples used for fertility preservation and predicting risks to future fertility in prepubertal males.

## Declaration of interest

The authors declare that the research was conducted in the absence of any commercial or financial relationships that could be construed as a potential conflict of interest.

## Funding

RTM was supported by a United Kingdom Research and Innovation (UKRI)https://doi.org/10.13039/100014013 Future Leaders Fellowship (MR/S017151/1). JBS was supported by a grant from the Swedish Childhood Cancer Fundhttps://doi.org/10.13039/501100006313 (TJ2020-0023). For the purpose of open access, the author has applied a Creative Commons Attribution (CC BY) licence to any Author Accepted Manuscript version arising from this submission.

## Author contribution statement

RTM, MT and GM conceived and designed the experiments. MT and GM performed the experiments. RTM and EK analysed the data. GF, JBS, DG, JD and SL provided reagents/materials/analysis tools/data/feedback on the manuscript. RTM and EK wrote the paper. All authors approved the manuscript.

## Data availability

The raw data supporting the conclusion of this article will be made available by the authors, without undue reservation.

## Ethics statement

The studies involving human participants were reviewed and approved by the South East Scotland Research Ethics Committee (LREC08/S1101/1), NRES Committee North East – Newcastle and North Tyneside 1 (08/H0906/21 + 5) and NRES Committee London – Fulham (18/10/0822), and written informed consent was given by the women. Ethical approval for the collection and use of human prepubertal testis tissues in research was obtained from the South East Scotland Research Ethics Committee (13/SS0145) and the Oxford University Hospitals NHS Foundation Trust (2016/0140). The patients/participants provided their written informed consent to participate in this study.
